# Genome-wide analysis of the SBT gene family involved in drought tolerance in cotton

**DOI:** 10.3389/fpls.2022.1097732

**Published:** 2023-01-11

**Authors:** Maohua Dai, Na Zhou, Yue Zhang, Yuexin Zhang, Kesong Ni, Zhenliang Wu, Liying Liu, Xiaoge Wang, Quanjia Chen

**Affiliations:** ^1^ Engineering Research Centre of Cotton, Ministry of Education/College of Agriculture, Xinjiang Agricultural University, Urumqi, China; ^2^ Dryland Farming Institute, Hebei Academy of Agricultural and Forestry Sciences/Hebei Key Laboratory of Crops Drought Resistance, Hengshui, China; ^3^ Institute of Industrial Crops, Shandong Academy of Agricultural Sciences, Jinan, China

**Keywords:** SBT, drought stress, VIGS, transmission electron microscopy, cotton

## Abstract

The subtilisin-like proteases (SBTs) are a large family of serine peptidases that are unique to plants. Previous studies have shown that SBTs are associated with developmental processes and environmental responses. However, comprehensive identification and systematic analysis of the SBT family have not been conducted in cotton. We used bioinformatics methods to analyze the structural characteristics, phylogenetic relationships, gene structures, expression modes, evolutionary relationships, selection pressures and stress responses of SBT gene family members in upland cotton. In this study, we identified 120 and 112 SBTs in the tetraploid cotton species G. hirsutum and G. barbadense, while 67 and 69 SBTs were identified in the diploid species G. arboreum and G. raimondii, respectively; these SBTs were divided into five distinct subfamilies. We identified the SBT gene GhSBT27A, and explore its function through virus-induced gene silencing and transmission electron microscopy. These results suggested that the GhSBT27A gene was involved in the response to drought stress. These results lay a foundation for further study on the drought stress mechanism of cotton.

## Introduction

Serine proteases are an important class of protein hydrolases with serine as the active center. Almost one-third of the proteases in living organisms are serine proteases (Von Groll et al., 2002; Page and Di Cera, 2008). Subtilisin-like proteases (SBTs) are serine proteases with catalytic triplets of aspartic acid, histidine and serine amino acids (Dodson and Wlodawer, 1998). SBTs are widely found in plants, bacteria, fungi and parasites (Siezen et al., 1991; Yamagata et al., 1994; Ksiazek et al., 2015; Jin et al., 2021). The conserved domains of SBTs are closely related to their multifunctional evolution in plants, and SBTs are widely involved in seed germination, cell division, tissue differentiation, seedling development, fruit ripening, plant senescence, the hypersensitive response (HR), programmed cell death, and apoptosis; SBTs also have diverse functions in the control of development, protein degradation and signal transduction (Arora and Singh, 2004; Antão and Malcata, 2005; Rautengarten et al., 2005; Galotta et al., 2019; Paulus et al., 2020).

In 1994, the first plant-based subtilisin protease was cloned in melon and named Cucumisin (Yamagata et al., 1994). Subsequently, an increasing number of SBTs have been separated, purified and identified from different plants. The SBT family has been thoroughly studied in the model crop *Arabidopsis thaliana*, with 56 SBTs, as well as some functions, have been identified (Rautengarten et al., 2005). *AtSBT6.1* and *AtSBT6.2* control cell elongation by processing the GOLVEN1 peptide (Ghorbani et al., 2016). *AtSBT3.8* is involved in the biogenesis of the bioactive PSK peptide, and overexpressing *AtSBT3.8* can improve the osmotic stress tolerance of transgenic plants (Stührwohldt et al., 2021). Moreover, it has also been reported that overexpression of *AtSBT4.13* could compensate for the inhibition of nitrogen oxides by PMA and improve acid tolerance (Bissoli et al., 2020). *AtSBT3.5* was found to be coexpressed with AtPME17 to control root growth (Sénéchal et al., 2014). In nonmodel plants, the results showed that overexpression of pineapple *AcoSBT1.12* could delay the flowering time of *Arabidopsis thaliana* under long-day conditions (Jin et al., 2021). In addition, knockdown of *TaSBT1.7* in wheat reduced the hypersensitivity response and resistance of wheat to stripe rust (Yang et al., 2021). These results suggest that SBTs not only play a role in plant-specific development but also participate in the plant response to environmental stress.

To further explore the biological functions of SBT gene families in plants, researchers systematically analyzed SBT gene families in different species, and 80, 63, 54, and 82 SBT gene family members were identified from *Vitis vinifera*, *Oryza sativa*, pineapple and *Solanum tuberosum*, respectively (Tripathi and Sowdhamini, 2006; Cao et al., 2014; Norero et al., 2016; Jin et al., 2021). Cotton has a large and complex genome. The identification and analysis of the SBT gene family in the cotton genome have not been reported.

Cotton is often affected by external environmental factors, such as drought, salinity, diseases and insect pests, during its growth and development. The harsh external environment can affect the growth of cotton and reduce the yield and fiber quality of cotton. Therefore, enhancing plant stress resistance and the immune system through genetic engineering is an effective and environmentally friendly means to improve plant resistance to stressful environments and pathogens. The completion of genome sequencing for four cotton species provides the basis for the comprehensive identification of SBTs in those species (Wang K. et al., 2012; Li et al., 2014; Hu et al., 2019). We also used bioinformatics to analyze the structural features, phylogenetic relationships, gene structures, expression patterns, evolutionary relationships, selection pressures and stress responses of the SBT gene family members in cotton. This study provides a new platform for functional genomic research and lays a foundation for further study on the drought stress mechanism of cotton.

## Materials and methods

### Cotton material and qRT-PCR analysis

A drought-tolerant upland cotton strain Zhong H177 was used as the experimental material. Plump seeds of similar size were selected and then planted in pots with heat sterilized sand. Cultures were incubated at 28°C/16 h light and 25°C/8 h dark and 75% relative humidity. When the third true leaf was fully expanded, cotton plants were divided into control and experimental groups. The control group was treated with clean water, while the experimental group was treated with 5% PEG6000. After 6 hours of treatment, leaves were collected from both groups of cotton and stored in an ultralow temperature refrigerator at −80°C for RNA extraction and qRT-PCR

assays. The primers for the *GhSBT27A* were as follows: forward primer, 5 ‘- CGTTCTATGCGATGTGATG- 3’, reverse primer, 5 ‘- GGTGGAATTGTGGTAGGA- 3’. qRT-PCR assays were performed on the Bio-Rad 7,500 fluorescence quantitative PCR platform with ChamQ Universal SYBR qPCR Master Mix (Vazyme Biotech Co.,Ltd,Nanjing, China) in accordance with the

manufacturer’s protocol. The experiments were independently repeated three times, and 2^-ΔΔCt^ method was used to measure relative gene expression levels.

### Identification of SBT gene family members

Whole genome sequence data for four cotton species, *Gossypium hirsutum* (ZJU), *Gossypium barbadense* (ZJU), *Gossypium arboreum* (CRI) and *Gossypium raimondii* (JGI), were obtained from the Cotton FGD (Cotton Functional Genomics Database) (https://cottonfgd.org/) (Zhu et al., 2017).

The hidden Markov pattern (HMM) map (https://pfam.xfam.org/) of peptidase S8 (PF00082), which most likely belongs to the subtilis protease gene family, was downloaded from Pfam. The protein sequence containing PF00082 was screened by HMMER software (Jones et al., 2014), and genes with incomplete domains were manually removed. Based on the location of the respective genes on the chromosome, we renamed the genes *GhSBT1A*-*GhSBT58A* and *GhSBT1D*-*GhSBT62D*. To understand the physicochemical properties of the *GhSBT* genes, we used the online tool Expasy-Protparam (https://web.expasy.org/protparam/) (Gasteiger et al., 2005). The subcellular localization of the SBT proteins was predicted using the online website WOLF-PSORT (https://wolfpsort.hgc.jp/).

### Construction of a phylogenetic tree of SBT family proteins

To investigate the evolutionary relationship among SBT genes, we performed multiple sequence alignments of the obtained genes using MEGA (MEGA7) and ClustalW (Kumar et al., 2016). Based on the comparison results, the evolutionary tree was constructed by the neighborhood method.

To explore the evolutionary relationship of SBT between the model crop *Arabidopsis thaliana* and four cotton species (*Gossypium arboretum, Gossypium raimondii, Gossypium hirsutum*, and *Gossypium barbadense*), homologous sequences of subtilisins from these species were obtained by the procedure described above. Multiple sequence alignments were performed using MEGA7 and ClustalW software, and interspecies evolutionary trees were constructed by the maximum likelihood method.

### Gene structure and conserved protein motif analysis of SBT family genes

To further understand the SBT family genes, we visualized the phylogenetic tree, conserved protein motifs and gene structure maps using the MAST file from MEME, the NWK file from the phylogenetic tree analysis and the GFF3 genome file from *Gossypium hirsutum* using TBtools software (Chen et al., 2020).

### Chromosomal locations and gene replication analysis of SBT genes

The whole genome annotation files of four cotton species, *Gossypium hirsutum* (ZJU), *Gossypium barbadense* (ZJU), *Gossypium arboreum* (CRI), and *Gossypium raimondii* (JGI), were obtained from the Cotton FGD (Cotton Functional Genomics Database) (https://cottonfgd.org/) (Zhu et al., 2017). MCScanX software was used to analyze genomic collinearity blocks. The physical chromosomal locations and gene replication of all SBT genes from the four cotton species were generated by TBtools software (Chen et al., 2020).

### Expression pattern and Cis-element analysis of SBT family genes

For analysis of differentially expressed genes, we have downloaded RNA-seq data (PRJNA248163), under different tissues and cold, heat, salt and PEG stresses from National Center for Biotechnology Information (https://www.ncbi.nlm.nih.gov/) (Hu et al., 2019).

To explore the regulation of gene expression, the 2.0 kb sequence upstream of the start codon was extracted from all the SBT family genes as promoter sequences for cis-element analysis. PlantCARE (Cis-Acting Regulatory Element) (http://:/bioinformatics.psb.ugent.be/webtools/PlantCARE/html/) was used to further analyze the cis-elements in the *GhSBT* gene promoter regions, and the cis-element information obtained was mapped using TBtools software (Chen et al., 2020).

### Collinearity analysis of SBT family genes

To explore the evolutionary relationship of SBT Family Genes in four cotton species, collinear genes were found throughout the genome, and all the cotton protein sequences were BLAST compared by MCSCANX software (Wang Y. et al., 2012). The results were visualized by TBtools software (Chen et al., 2020).

### Selective pressure calculation

To investigate the selective pressures on SBT genes during evolution, we used TBtools software to calculate the rates of nonsynonymous substitutions (Ka) and synonymous substitutions (Ks) for duplicate genes (Chen et al., 2020).

### VIGS technology silencing of *GhSBT27A*

To study the function of the subtilis enzyme genes in the drought tolerance of cotton, one SBT gene (the highly expressed *GhSBT27A*) was silenced by virus-induced gene silencing. The VIGS vector was the laboratory storage vector pYL156, and we constructed pYL156:*GhSBT27A* with restriction enzyme cleavage sites XbaI and BamHI. The primers for the *GhSBT*-silencing fragment were as follows: forward primer, 5 ‘- aaggttaccgaattctctagaTTAATCAAAAGTTATAAAAGGAGCTTCA - 3’, reverse primer, 5 ‘- cgtgagctcggtaccggatccGGCTGCTGTGGATGCCGT - 3’. The VIGS (virus-mediated gene silencing) system consisted of the recombinant vector pYL156:*GhSBT27A*, positive control pYL156:PDS, negative control pYL156 and auxiliary vector pYL192. When plants reached the three-leaf stage, the control group was irrigated with aqueous solution, while the experimental group was irrigated with the same volume of 5% PEG6000.

### Transmission electron microscopy

To investigate the effect of genes on the morphology of cotton cells, we used transmission electron microscopy to observe the morphological changes to chloroplasts in leaves of gene-silenced cotton plants. Inverted second leaves of the same size were selected for the control and treatment groups. The leaf tissues (1 mm^3^) were kept fresh to minimize mechanical damage such as pulling, bruising and crushing of the tissue and to minimize the sampling time. A Petri dish with electron microscope fixative was prepared before sampling, and the small tissue pieces were put into the Petri dish immediately after removal and cut into 1 mm^3^ pieces with a scalpel. The cut tissue blocks were then transferred to EP tubes with new electron microscope fixative for further fixation and pumped by vacuum until they sank to the bottom. Samples were left at room temperature for 2 h and then stored at 4°C. After postfixation, dehydration and permeation embedding, the samples were inserted into embedding plates and placed in an oven overnight at 37°C. The samples were washed three times with 0.1 M phosphate buffer PB (pH 7.4) for 15 min each time. We used Image-Pro Plus 6.0 (Media Cybernetics, Inc., Rockville, MD, USA) as our transmission electron microscopy data analysis software.

### Subcellular localization of *GhSBT27A*

To observe the subcellular localization of *GhSBT27A*, the constructed vector plasmid was transferred into Agrobacterium GV3101 and cultured for 2 d at 30°C. The suspended bacterial solution was injected into the tobacco leaves, cultured for 2 d under low light condition, and well labeled. The tobacco leaves in the injection area were made into glass slides, observed and photographed under a confocal microscope (Zeiss LSM 710). Empty GFP vector without *GhSBT27A* gene was used as control.

## Results

### Identification of SBT gene family members in cotton

A latent Markov model of Peptidase_S8 (PF00082) was obtained from domain prediction and used to identify members of the SBT gene family in the whole cotton genome, as well as for comparison with the CDD, SMART, and other databases; genes with incomplete domains were removed manually. A total of 120 pairs of genes belonging to the SBT gene family were identified. The genes were renamed *GhSBT1A-GhSBT58A* and *GhSBT1D-GhSBT62D* according to their position on the chromosome. Then, analysis of the physical and chemical properties of the amino acid sequence of cotton SBT gene family members was performed (**Supplementary Table S1**). The results showed that the molecular weight of 120 SBT genes ranged from 15.289 to 226.819 kDa. All identified SBT genes encode proteins with amino acid lengths in the range of 136 to 2111, with an isoelectric point range of 4.387 to 10.369, with an average of 7.382, suggesting that these proteins were weakly alkaline. Subcellular localization predicted 56 genes in the chloroplast, 20 genes in the extracellular space, 13 genes in the vacuole, 9 genes in the plasma membrane, 8 genes in the cytoplasm, 8 genes in the endoplasmic reticulum, 4 genes in the nucleus, 1 gene in the cytoskeleton, and 1 gene in the mitochondrion. The subcellular localization results demonstrate that members of the *GhSBT* family play key roles in numerous biological processes, including plant growth and development.

### Phylogenetic analysis of *SBTs*

**Figure 1:**
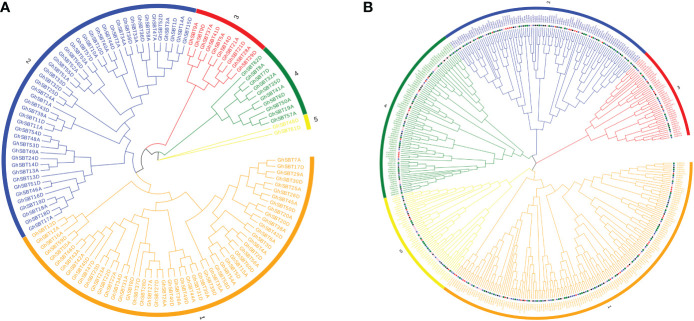
Two unrooted phylogenetic trees of SBT genes were constructed by MEGA7: the evolutionary tree of the *GhSBT* family was constructed using the neighbor-joining method, and the interspecific evolutionary tree of SBTs was constructed using the maximum likelihood method. **(A)** Phylogenetic tree of the SBT family protein sequences in upland cotton; **(B)** Phylogenetic relationships of 422 SBT proteins from *G. hirsutum, G. barbadense, G. arboreum, G. raimondii*, and *Arabidopsis*.

To analyze the evolutionary relationship between each member of the SBT family, ClustalW in MEGA7 software was used to compare 120 SBT protein sequences, and a rootless phylogenetic tree was constructed by the adjacency method (**Figure 1A**). As shown in **Figure 1A**, the SBT protein sequences are divided into five distinct subfamilies. The group marked in orange is the largest subfamily, SBT1, which contains 52 SBT genes. SBT2-SBT5 correspondingly contain 46, 10, 10 and 2 SBT genes. Genes from the same subgroup can be considered to have the same or similar functions. SBT proteins from homologous chromosome subgroups A and D are mostly clustered in the same branch.

To better elucidate the phylogenetic relationship between the four cotton species and the Arabidopsis SBT genes, we used 422 protein sequences from *G. hirsutum* (120), *G. barbadense* (112), *G. arboreum* (67), *G. raimondii* (69), and *Arabidopsis* (54) to construct an evolutionary tree (**Figure 1B**). Based on the latest TAIR database, the *Arabidopsis* SBT protein sequences are slightly different from those previously reported, with the deletion of *At5g58810* and *At4g20850* due to incomplete structural domains. The SBT proteins of these species are distributed in almost every branch. The phylogenetic tree was randomly divided into five subclades. Among these branches, subclade SBT3 had the fewest members (42), subclade SBT1 had the most members (153), and subclades SBT2, SBT4, and SBT5 contained 97, 81, and 49 genes, respectively. Notably, most of the SBT proteins in Arabidopsis and the four cotton species have corresponding homologous proteins in all subclades, suggesting that SBT proteins in these plants may be functionally conserved in dicotyledons. In addition, it has previously been shown that upland cotton and island cotton are the result of crosses between *Gossypium arboretum* and *Gossypium raimondii*. This was also confirmed by our finding that SBT proteins in tetraploid cotton (*Gossypium hirsutum* and *Gossypium barbadense*) and diploid cotton (*Gossypium arboretum* and *Gossypium raimondii*) always cluster together. In addition, the *GhSBT* and *GbSBT* pairs always cluster together, which also shows the importance of gene duplication during evolution.

### Chromosomal location of SBTs in four *Gossypium* species

**Figure 2:**
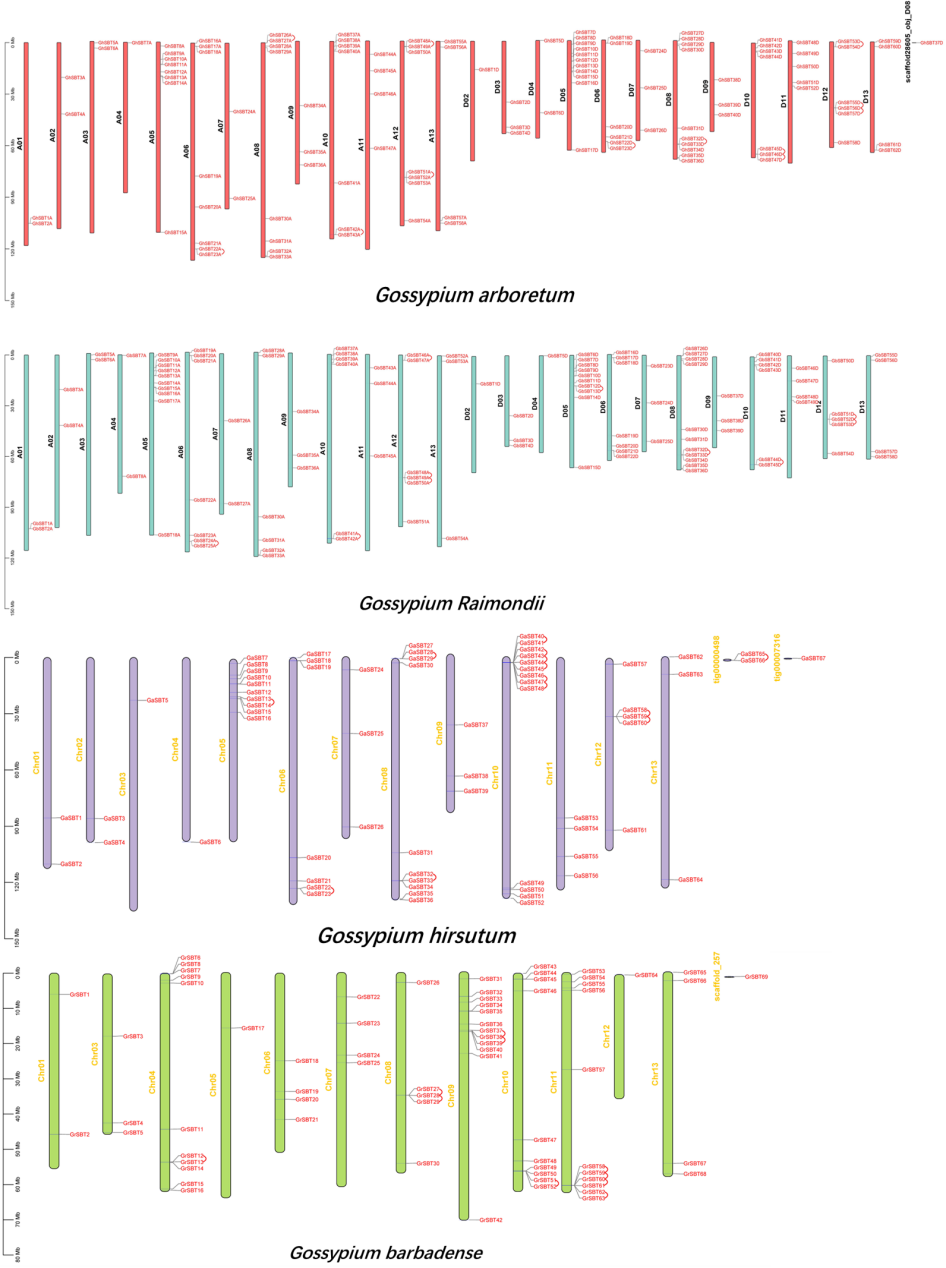
Chromosomal localization and gene duplication of SBT genes in *Gossypium arboretum*, *Gossypium raimondii*, *Gossypium hirsutum*, and *Gossypium barbadense*, and tandem duplication of gene pairs during evolution is shown by lines.

To further study the chromosomal distribution and gene replication of SBT genes in four Gossypium species, we mapped the physical locations of these genes on chromosomes (**Figure 2**). The 368 genes were randomly distributed on specific chromosomes of the four *Gossypium* species. In *Gossypium hirsutum*, 119 genes were randomly distributed on 25 chromosomes, and 1 gene was localized on the scaffold. The number of SBT genes on each chromosome was between 1 and 11. Tandem replication occurred on chromosomes A06, A08, A10, and A12 and D06, D08, D10, and D12. There were 58 genes in the A subgenome and 62 genes in the D subgenome. There is no SBT gene on chromosome D01, which may be because these predicted SBT genes might have been duplicated or lost during evolution. In *Gossypium barbadense*, 112 genes were randomly distributed on 25 chromosomes, and there was no SBT gene on chromosome D01, similar to *Gossypium hirsutum*, which supports the gene duplication. The number of SBT genes on each chromosome was between 1 and 11. There were 54 genes in the A subgenome and 58 genes in the D subgenome. These results are similar to those for *Gossypium hirsutum*, indicating that the genetic evolutionary process of SBT genes is mature and stable. Tandem replication occurred on chromosomes A06, A10, and A12 and D05, D08, D10, and D12. In *Gossypium arboretum*, 64 genes were distributed on 13 chromosomes, and 3 genes were distributed on the scaffold, all of which were unevenly distributed. The number of SBT genes on each chromosome was between 1 and 13, there was at least 1 gene on chromosome A03 and up to 13 genes on chromosome A10, and tandem replication occurred on Chr05, Chr06, Chr08, Chr10, and Chr12 and 1 scaffold, tig00000498. In *Gossypium raimondii*, 68 genes were distributed on 12 chromosomes, and 1 gene was distributed on the scaffold, all of which were unevenly distributed. The number of SBT genes on each chromosome was between 1 and 12, there was no SBT gene on chromosome D02, and tandem replication occurred on Chr04, Chr08, Chr09, Chr10, and Chr11. In summary, both tandem and fragmental duplication are the main modes of gene amplification during the evolution of SBT genes.

### Correlation analysis of *GhSBT* gene structure and motif composition

**Figure 3:**
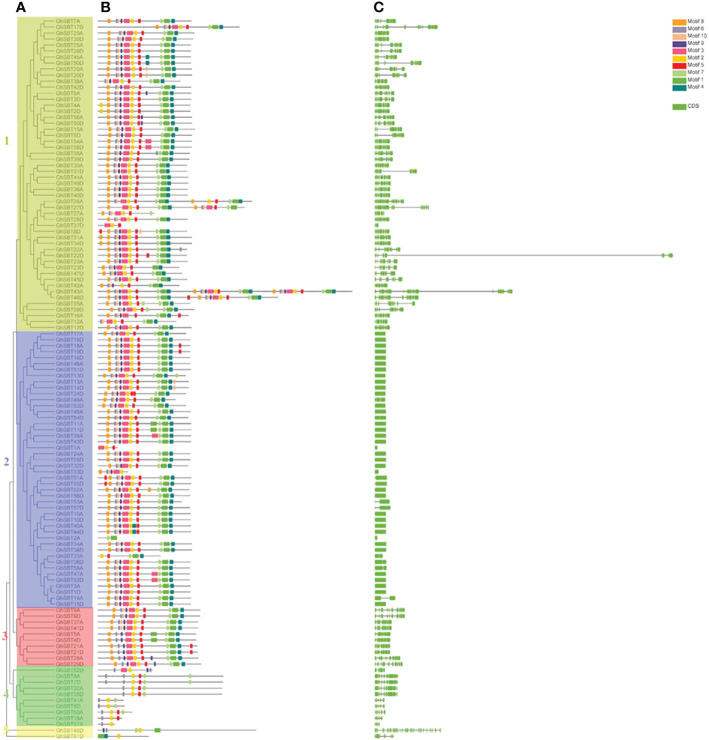
Genetic structure of the SBT gene family in upland cotton. **(A)** Phylogenetic tree of the SBT gene family. **(B)** Motif pattern diagram of the SBT gene family. **(C)** Exon structure diagram of the SBT gene family.

To further understand the possible structural evolutionary relationships of *GhSBT* family members, phylogenetic trees, motif association analysis and gene structure analysis of *GhSBT* genes were performed (**Figure 3**). The protein sequences and annotation files of *GhSBT* members were used to construct phylogenetic trees and gene structure information. MEME and TBtools software were used to analyze the conserved motifs in SBT proteins (**Figures 3A**, **B**). Ten putative motifs were identified in the *GhSBT* members. The number of motifs varied for each family member, ranging from 3-30, and members of the same subgroup shared a similar motif composition. The differences in motifs may represent diversity in their functions. The subfamilies SBT1 and SBT2 contain almost all motifs, except *GhSBT27A*, *GhSBT37D*, *GhSBT1A*, *GhSBT33D*, *GhSBT2A*, and *GhSBT33A*. Some members of subfamily SBT3 do not contain motif 10. The N terminus of all members of subfamily SBT4 starts with motif 6 and contains motif 2. Members of subfamily SBT5 contain only a few motifs, and presumably mutations have occurred during evolution.

To further explore the diversity of SBT gene structures, the characteristics of intron-exon structures were analyzed. As shown in **Figure 3C**, the same subfamily has similar intron-exon arrangements, and the structures of SBT genes can be classified into two types, those with fewer introns and those with more exons. Members of the subfamily SBT2 contain fewer exons, basically ranging from 1-2, while other subfamily members have more exons, ranging from 3 to 34.

### *GhSBT* expression patterns and promoter analysis

To better investigate the mechanisms of gene regulation, we identified the cis-acting elements on each gene studied; these elements can be used to study different environmental stress reactions and tissue specificities.

**Figure 4:**
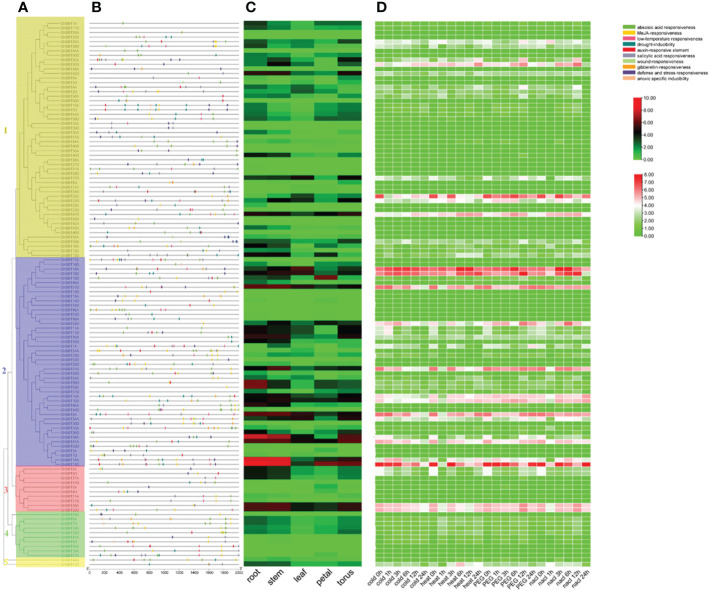
Analysis of promoters and differentially expressed genes of the *GhSBT* family. **(A)** Phylogenetic tree of *GhSBT* genes. **(B)** Cis-acting elements in the promoters of *GhSBT* genes. **(C)** The organizational expression of *GhSBT* genes. **(D)** The expression patterns of *GhSBT* genes under cold, hot, PEG and salt stresses.

We used the online tool PlantCARE to predict the promoter region 2000 bp upstream of *GhSBT* genes. Among them, the cis-acting elements related to plant hormones included abscisic acid response elements, salicylic acid response elements, gibberellin response elements, MeJA response elements and auxin response elements. The selected abiotic stress response factors included defense and stress response factors, trauma response factors, drought inducing factors and low temperature response factors. Almost all promoters contain several hormone response elements, but the hormone response elements are not closely related to their subfamilies (**Figures 4A**, **B**). Most promoters from *GhSBT* members contain ABA response elements, MeJA regulatory elements, and salicylic acid regulatory elements. We found that 76 genes contained abscisic acid-responsive elements, 77 genes contained MeJA-responsive elements, 59 genes contained salicylic acid-responsive elements, 20 genes contained gibberellin-responsive elements, and 36 genes contained auxin-responsive elements. In addition, we identified a large number of response components for abiotic stresses, such as 53 genes containing defense and stress-responsive elements, 50 genes containing drought-inducible elements, 38 genes containing low-temperature responsive elements, 9 genes containing wound-responsive elements and 6 genes containing anoxic-specific inducible elements. Through promoter analysis, we can summarize the genes that respond to different plant hormones and reaction mechanisms under different stresses, which will help us to validate the subsequent gene functions.

To clarify the mechanism of the response of *GhSBTs* to abiotic stress, we used RNA-seq to analyze genes differentially expressed under cold, heat, salt and PEG stresses. The results showed that gene expression changed under different stresses, suggesting that members of *GhSBTs* are involved in the regulation of abiotic stresses (**Figure 4D**). We found that genes from the same branch mostly had the same expression pattern. Of interest, some genes were highly expressed under specific stresses; for example, *GhSBT1A* was strongly induced under PEG stress at 24 h and not under other stresses; *GhSBT61D* was only induced under heat stress. In conclusion, *GhSBT* gene expression levels changed under different stresses, guessing that these genes play an important role in the response to abiotic stresses. In parallel, to explore the tissue expression specificity of *GhSBTs*, we used their expression data (FPKM values) in different tissues (root, stem, leaf, petal, and torus) and generated a heatmap (**Figure 4C**). The results showed that *GhSBT2A*, *GhSBT14A*, *GhSBT58A*, and *GhSBT15D* were highly expressed in roots and stems, and most of the genes were tissue specific.

### Collinearity analysis

Gene family evolution mainly includes whole genome replication, fragment replication and tandem replication (Xu et al., 2012). Most plants underwent an ancient genome-wide replication event, or polyploidy, resulting in the duplication of all genes in a region. This large-scale chromosomal doubling event resulted in the retention of a large number of chromosomal doubling fragments in the genome (Malik et al., 2020). Tandem repeats occur on the same chromosome and are adjacent to each other, often with similar sequences and similar functional clusters (Xing et al., 2011). Fragment duplications are duplicated genes that are located far apart or on different chromosomes. Gene duplication events are the main cause of gene family expansion and doubling and have an important impact on evolutionary dynamics (Chothia et al., 2003).

**Figure 5:**
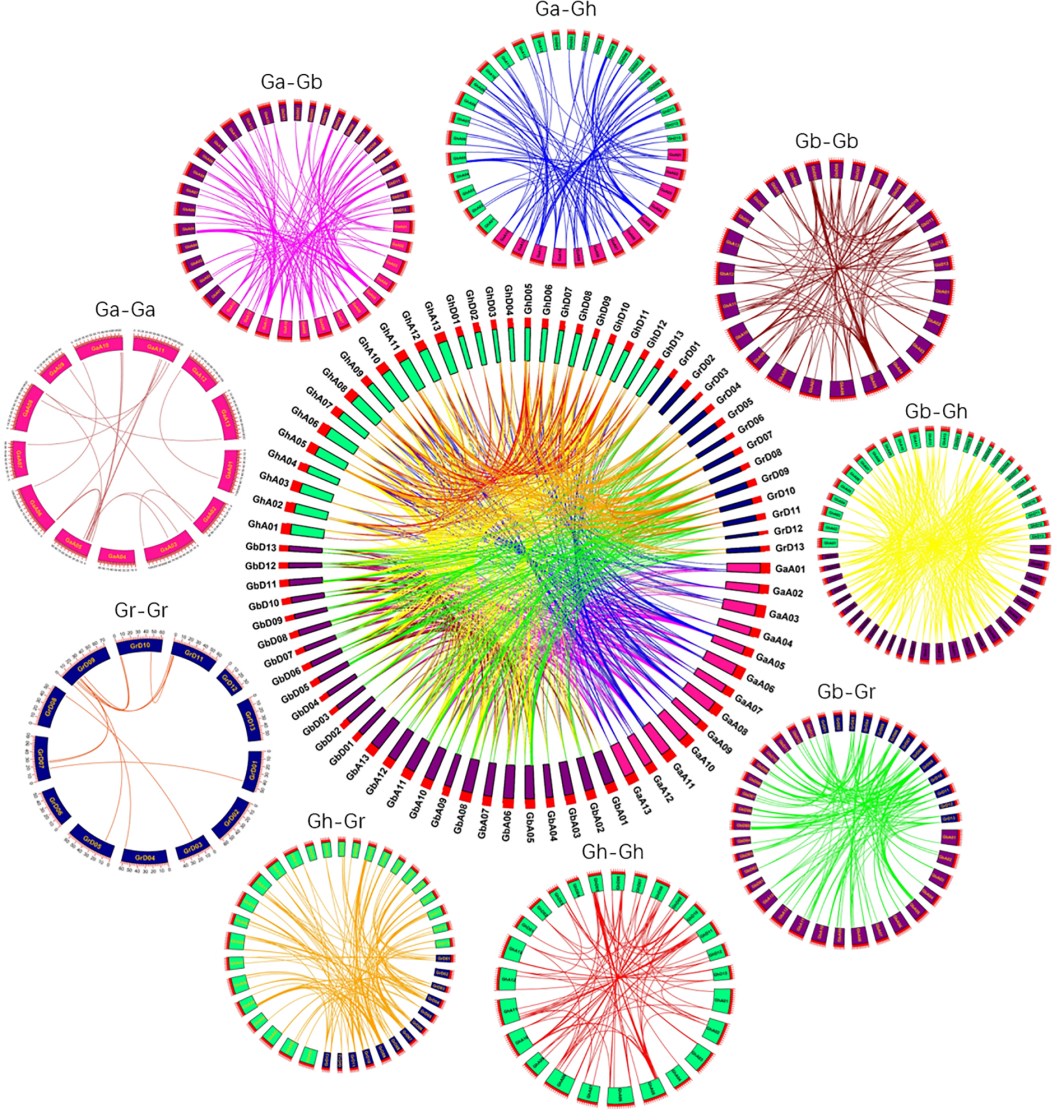
Collinearity of repeated gene pairs in four cotton specie*s* (*Gossypium hirsutum, Gossypium barbadense, Gossypium arboreum* and *Gossypium raimondii*). The collinearity region around the SBT gene is indicated by chromosomal lines of different colors.

Through the homology analysis of SBT genes in four cotton varieties (*Gossypium arboretum, Gossypium raimondii, Gossypium hirsutum*, and *Gossypium barbadense*), we visualized the relationships between SBT genes from the four cotton varieties (**Figure 5**). Large-scale whole-genome duplications (WGDs) and small-scale tandem duplications, as well as fragment duplications, between species can be identified from the collinear fragments; these can be used as the basis for species tree inference. In **Figure 5**, the same genes are connected by lines of the same color. By comparing the genomes and subgenomes of Ga-Ga, Ga-Gb, Ga-Gh, Gb-Gb, Gb-Gr, Gb-Gh, Gr-Gh, Gr-Gr and Gh-Gh, we identified a total of 1313 pairs of linear/paralogous gene pairs, where 45 duplicated gene pairs showed tandem duplication (**Figure 2**). A total of 269 pairs of duplicated genes underwent fragment duplication, and the remaining 999 pairs of duplicated genes underwent whole-genome duplication. Among them, there were 21, 130, 105 and 13 collinear gene pairs with fragment duplication in Ga-Ga, Gb-Gb, Gh-Gh and Gr-Gr, respectively. There were 136, 173, 338, 186 and 166 linear/paralogous gene pairs replicated in Gh-Ga, Gh-Gr, Gh-Gb, Gb-Gr and Gb-Ga, respectively (**Supplementary Table S2**). Therefore, we hypothesized that the major causes of gene amplification during SBT gene evolution were whole-genome duplication events and fragment duplication events.

### Calculation of non−synonymous (Ka) to synonymous (Ks) substitution rates

To study the phylogeny and understand the relatedness of protein-coding sequences, we performed selective pressure analyses. The ratio between the nonsynonymous substitution rate (Ka) and synonymous substitution rate (Ks) of two protein-coding genes was calculated to determine whether there was selective pressure acting on them. Synonymous mutations are not thought to be subject to natural selection because they do not affect amino acid sequences or protein structure or function. Nonsynonymous mutations, on the other hand, are subject to natural selection because they can affect amino acid sequences and may alter protien structure and function. Therefore, if Ka/Ks > 1, it is considered that there is positive selection effect. If Ka/Ks = 1, neutral selection is considered to occur. If Ka/Ks < 1, a negative selection effect is considered to be present, i.e., a purification effect or purifying selection.

**Figure 6:**
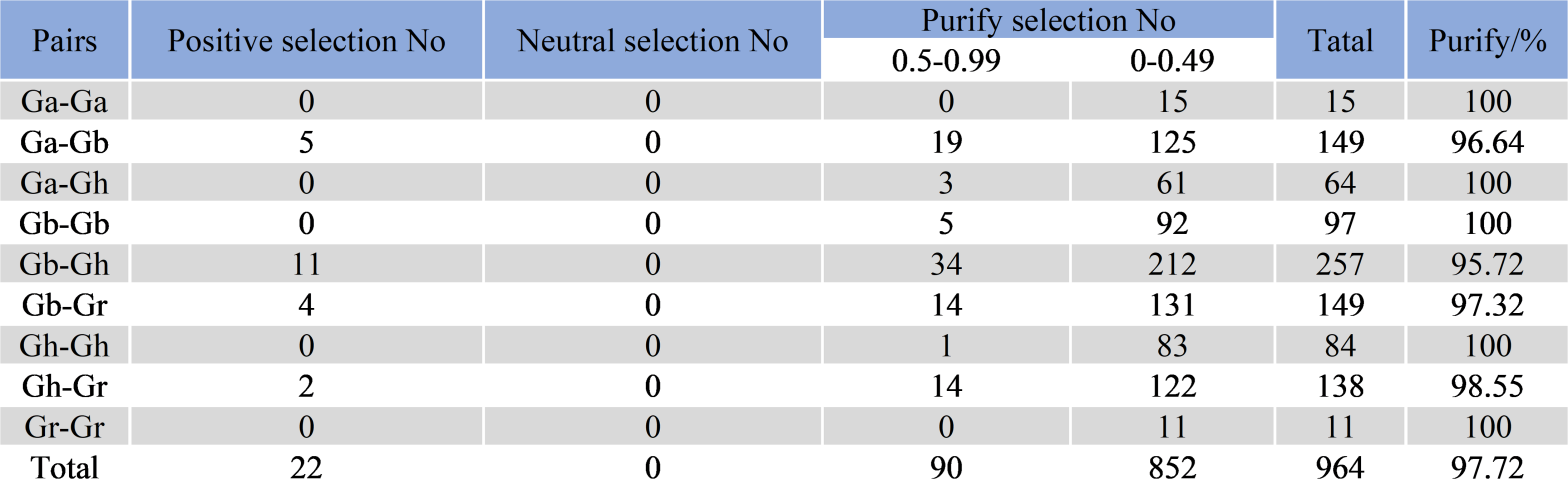
Prediction of no of duplicated gene pairs involved in different combinations from four *Gossypium* species.

The Ka/Ks values of 964 gene pairs in 4 cotton species were calculated by TBtools software (**Figure 6**), and 22 pairs of genes had Ka/Ks values greater than 1, indicating that these genes were rapidly evolving in recent years and may have had great significance for the evolution of species. There were 852 pairs of genes with Ka/Ks values between 0 and 0.5, and 90 pairs of genes with Ka/Ks values between 0.5 and 0.99, of which 97.72% had Ka/Ks values less than 1 (**Supplementary Table S3**). This suggests that SBTs have undergone intense purifying selection during evolution.

### Expression and silencing analysis of *GhSBT27A* under PEG stress in cotton

**Figure 7:**
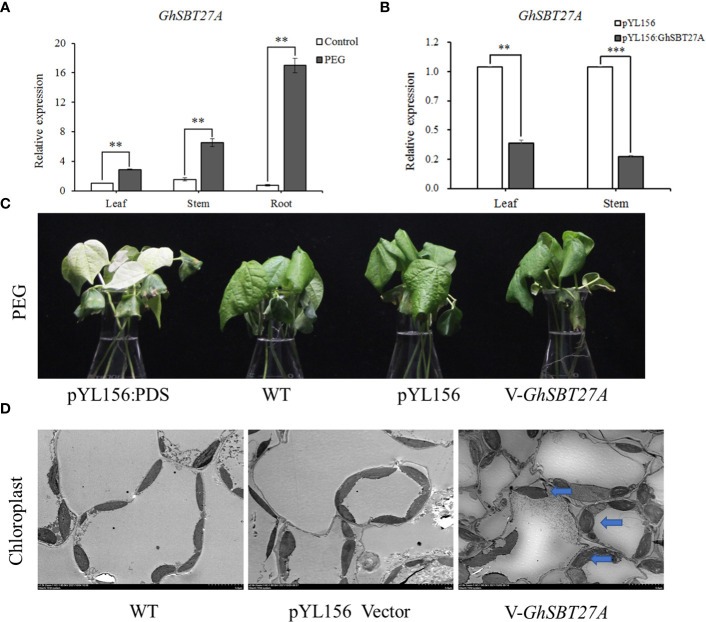
Functional verification of *GhSBT*. **(A)** Relative expression of *GhSBT27A* in different tissues. **(B)** Detection of *GhSBT27A* silencing efficiency. **(C)** Phenotypic comparison of *GhSBT27A*-silenced plants under PEG stress. **(D)** Comparison of chloroplast morphology in *GhSBT27A*-silenced plants under PEG stress as observed by transmission electron microscopy. **and ***represent the differences between the three tissues of cotton roots, stems and leaves at p < 0.01 and p < 0.001 respectively.

To further understand the function of SBT gene family members in the drought resistance of upland cotton, we selected a highly expressed gene, *GhSBT27A*, from our PEG stress transcriptome data for further study. To investigate the effect of *GhSBT27A* on PEG stress, we detected the relative expression levels of *GhSBT27A* in the roots, stems and leaves under PEG stress(**Figure 7A**). The results showed that *GhSBT27A* in different tissues were up-regulated after PEG treatment, the relative expression level of *GhSBT27A* in roots under PEG is the highest. Furthermore, we reduced the expression of endogenous *GhSBT27A* in cotton. The success of the VIGS experiment was confirmed when the albino phenotype was observed for pYL156:PDS cotton plants (**Figures 7B**, **C**). The expression levels of *GhSBT27A* in the stems and leaves of wild-type, pYL156, and pYL156:*GhSBT27A* cotton plants were determined by qRT-PCR. As shown in **Figure 7B**, the relative expression levels of *GhSBT27A* in wild-type plants and pYL156 plants were basically the same in different tissues of cotton. The relative expression level of *GhSBT27A* in cotton plants injected with pYL156:*GhSBT27A* vector was significantly lower than that in control cotton plants injected with pYL156 (empty vector), indicating that the gene was successfully knocked down. The above cotton plants were treated with a 5% concentration PEG. After 6 h of treatment, *pYL156:GhSBT27A* plants exhibited a more severe wilting phenotype, while control plants (PEG-pYL156) were relatively less affected (**Figure 7C**). To further verify the effect of *GhSBT27A* on the changes in leaf tissue cells when cotton responded to PEG stress, we simultaneously treated gene-silenced cotton plants, wild-type cotton plants and cotton plants carrying pYL156 with 5% PEG, and their leaf tissue cells were observed and analyzed by transmission electron microscopy. The results are shown in **Figure 7D**. Compared with wild-type and empty vector-carrying cotton plants, *GhSBT27A* gene-silenced cotton plants showed degraded chloroplast envelopes, and the overall appearance was ellipsoid. Separation was also more frequent. These results suggest that *GhSBT27A*-silenced plants are more sensitive to PEG and have reduced drought tolerance.

### Subcellular localization analysis

**Figure 8:**
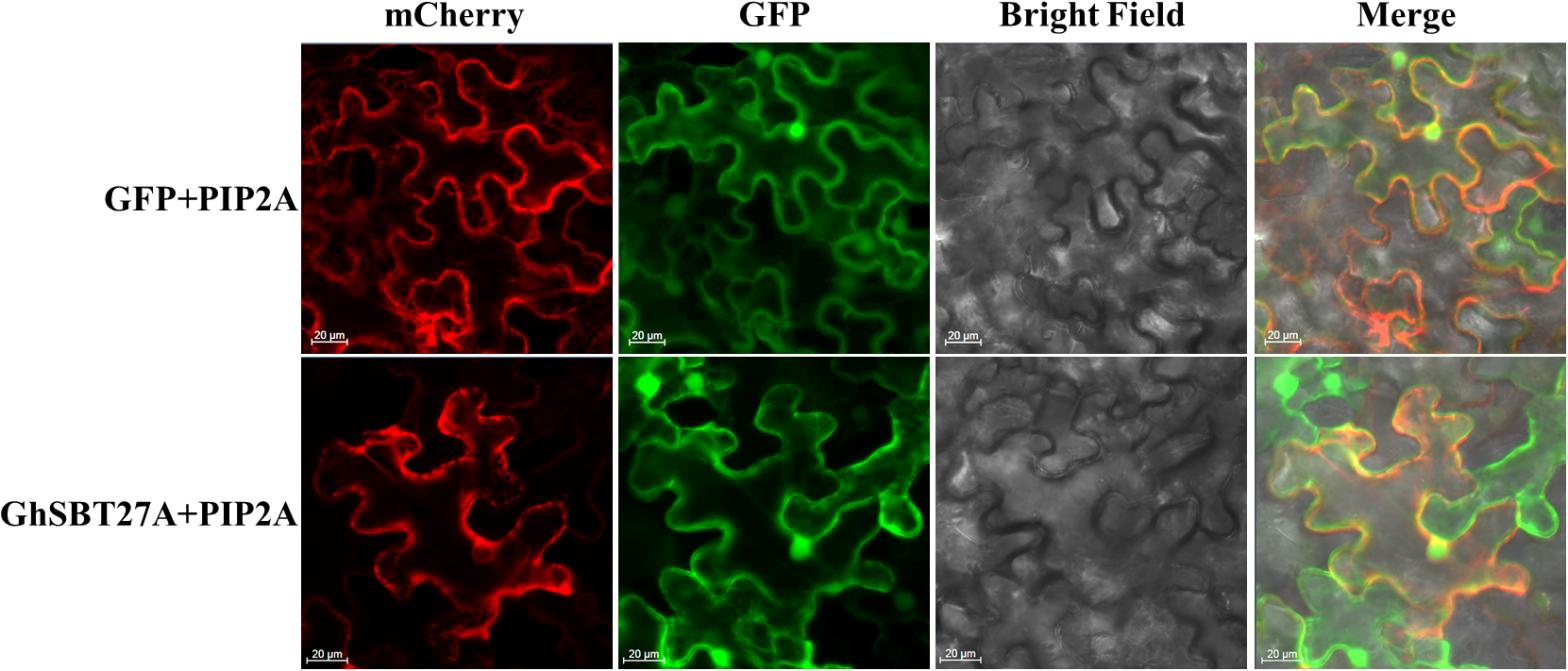
Subcellular localization of *GhSBT27A* in tobacco leaves.

To confirm the location of *GhSBT27A* in the cell, GFP- *GhSBT27A* and GFP alone were transiently expressed in tobacco epidermal cells (**Figure 8**). In tobacco cells expressing GFP protein alone, fluorescence occurs in the cell membrane, cytoplasm and nucleus, whereas the GFP fluorescence of *GhSBT27A* was present on the membrane and nucleus. The results indicate that the membrane and nucleus are the major distribution locations of *GhSBT27A*.

## Discussion

Due to the prevalence of water-scarce environments and a changing climate, drought is a frequent problem during the crop growing season, greatly limiting agricultural production, and it is the most serious environmental factor limiting global crop yields (Barkla et al., 2013; Zhang et al., 2018). Cotton is an important cash crop and oilseed crop in China. It is particularly important to improve the tolerance of cotton varieties to drought and other stresses (Meshram et al., 2022).

SBTs are widely found in plants, bacteria, fungi and parasites. The conserved domain of SBT is closely related to its multifunctional evolution in plants and is widely involved in control of development, protein degradation and signaling. Genome-wide analysis of the SBT gene family has been performed in *Arabidopsis thaliana*, *Vitis vinifera*, *Oryza sativa*, pineapple and *Solanum tuberosum* (Tripathi and Sowdhamini, 2006; Cao et al., 2014; Norero et al., 2016; Jin et al., 2021). The genome of cotton is large and complex. The identification and analysis of the SBT gene family in the cotton genome has not been previously reported. The completion of genome sequencing for four cotton species provides a basis for the comprehensive identification of SBT genes in those species (Paterson et al., 2012; Hu et al., 2019).

Based on published information on the upland cotton (TM-1) genome, 120 SBT genes were identified by BLASTP. These genes encode proteins with 136-2,111 amino acids, a molecular weight of 15.289-226.819 kDa, and an isoelectric point of 4.387 to 10.369, with an average of 7.382, suggesting that these proteins are weakly basic. The members of the SBT family encode proteins with different physicochemical properties and different functions and regulatory mechanisms, but they all share a stable SBT structural domain (Xie et al., 2018). The results of subcellular localization showed that most of these proteins were localized to chloroplasts, including *GhSBT27A*, which also coincided with the results of our electron microscopy experiments, suggesting that this gene may further influence drought tolerance in plants by affecting the structural integrity of chloroplasts. These subcellular localization results suggest that members of the *GhSBT* family may play key roles in many biological processes, including plant growth and development.

In this study, we identified and analyzed the SBT family genes of five species, including *Gossypium hirsutum*, *Gossypium barbadense*, *Gossypium arboreum*, *Gossypium raimondii*, and *Arabidopsis thaliana*. An evolutionary tree was constructed based on their evolutionary relationships. The evolutionary tree divided these genes into five branches, each of which contained genes for SBTs present in all five species. It was shown that SBT genes are present not only in cotton and *Arabidopsis* but also in other monocotyledonous species, indicating that SBT genes are present in the genomes of both dicots and monocots. This means that the SBT gene family formed subgroups I, II, III, IV and V before the separation of dicots and monocots, and it has also undergone different periods of differentiation. Analysis of the gene structures and conserved motifs of the SBT family members showed that these five branches have similar gene structures and conserved motifs (Xie et al., 2018). The presence of a large number of genes in cotton demonstrated that the SBT family has an important and stable function. In addition, collinearity analysis showed that a large number of tandem repeats occurred between homologous chromosomes. These results suggest that modes of gene family expansion, including duplication, and multiple gene copies prevent gene mutation-induced gene function loss, which indicates the importance of their function (Xu et al., 2012; Zhang et al., 2022). Among these genes, *GhSBT26* and *GhSBT27A* are tandemly repeated genes. Tandem, segmental and whole genome replication play a crucial role in the expansion of gene families.

In terms of chromosomal location, SBT genes were unevenly distributed in the At and Dt subgroups. However, the distribution patterns converged in both subgenomes, with both having SBT genes present on most chromosomes. Most of the SBT genes were distributed at both ends of the chromosome, while a few genes were distributed in the middle. The stable heritability of these genes indicates the importance and wide range of functions for the SBT gene family (Cannon et al., 2004).

We calculated the Ka/Ks ratio of 964 gene pairs to investigate the influence of selective pressure on SBT genes during evolution. Among them, there were 942 duplicate gene pairs with Ka/Ks < 1, indicating purifying selection. There were 22 genes with Ka/Ks > 1, which indicated that these genes were rapidly evolving in recent years and had very important significance for the evolution of the species. In the cotton SBT family, 97.72% of the gene pairs underwent purifying selection, indicating that strong purifying selection occurred after tandem duplication, fragment duplication and whole genome duplication in this gene family. However, the selection pressure of most gene pairs was between 0 and 0.49, indicating that SBT gene pairs tended to be conserved during evolution (Panchy et al., 2016).

When plants are subjected to abiotic stresses, cis-acting elements upstream of each gene play an important role. These cis-acting elements do not encode proteins, but they can regulate gene expression (William Roy and Gilbert, 2006; Morello and Breviario, 2008). The cis-acting element prediction results showed that most of the upland cotton *GhSBTs* genes were associated with various stresses, such as drought, low temperature, and defense, as well as hormone responses, such as responses to abscisic acid, gibberellin, salicylic acid, growth hormone, and MeJA. This result indicates that *GhSBTs* are not only involved in multiple signaling pathways but also in plant growth and development and defense responses, providing a reference for screening for stress resistance genes (Nakashima et al., 2014). The *GhSBT27A* gene contains multiple abscisic acid response elements, which are consistent with our findings that this gene regulates drought resistance in upland cotton.

Based on the results of our study, we hypothesized that *GhSBT27A* may affect plant drought tolerance by affecting chloroplast structure. Our data showed that under PEG stress, the relative expression levels of *GhSBT27A* were up-regulated, the relative expression level of *GhSBT27A* in roots under PEG is the highest. We used virus-induced gene silencing to silence *GhSBT27A*, and under PEG stress, *GhSBT27A*-silenced plants were more severely stressed than the control, suggesting that the *GhSBT27A* gene plays an important role in drought stress resistance in cotton. Moreover, transmission electron microscopy results showed that chloroplast degradation was more severe in *GhSBT27A*-silenced plants than in control plants, which further suggested that the *GhSBT27A* gene might resist drought stress by regulating chloroplast morphological structure.

## Conclusion

This study provides the first comprehensive analysis of the SBT gene family in cotton. Here, 368 SBT genes were detected in four cotton species, including 120 *GhSBTs* in upland cotton, which underwent tandem and genome-wide duplication during evolution. *GhSBTs* were classified into five branches based on the phylogenetic tree, gene structure and motif analyses. The study of *GhSBT27A*, a member of evolutionary branch 1, revealed that it plays an important role in regulating drought stress in cotton. This study enriches the understanding of upland cotton SBT genes and lays the foundation for further studies on the functions of *GhSBTs* in cotton.

## Data availability statement

Publicly available datasets were analyzed in this study. This data can be found here: https://cottonfgd.org/, https://pfam.xfam.org/.

## Author contributions

QC designed the project. MD, NZ and YZ conducted the experiments. MD wrote the manuscript. NZ and YXZ performed the bioinformatics analysis. NZ, YXZ, KN, ZW, LL and XW assisted in writing and editing. QC was responsible for revising the manuscript. All authors have read and approved the final manuscript. All authors contributed to the article and approved the submitted version.
